# Effect of Alcohol on Hippocampal-Dependent Plasticity and Behavior: Role of Glutamatergic Synaptic Transmission

**DOI:** 10.3389/fnbeh.2019.00288

**Published:** 2020-01-24

**Authors:** Rodrigo G. Mira, Matias Lira, Cheril Tapia-Rojas, Daniela L. Rebolledo, Rodrigo A. Quintanilla, Waldo Cerpa

**Affiliations:** ^1^Laboratorio de Función y Patología Neuronal, Departamento de Biología Celular y Molecular, Facultad de Ciencias Biológicas, Pontificia Universidad Católica de Chile, Santiago, Chile; ^2^Laboratory of Neurobiology of Aging, Universidad San Sebastián, Santiago, Chile; ^3^Laboratory of Neurodegenerative Diseases, Universidad Autónoma de Chile, Providencia, Chile; ^4^Centro de Excelencia en Biomedicina de Magallanes (CEBIMA), Universidad de Magallanes, Punta Arenas, Chile; ^5^Escuela de Obstetricia y Puericultura and Centro Integrativo de Biología y Química Aplicada (CIBQA), Facultad de Salud, Universidad Bernardo O Higgins, Santiago, Chile

**Keywords:** alcohol dependence, hippocampus, plasticity, glutamatergic synaptic transmission, neuronal toxicity

## Abstract

Problematic alcohol drinking and alcohol dependence are an increasing health problem worldwide. Alcohol abuse is responsible for approximately 5% of the total deaths in the world, but addictive consumption of it has a substantial impact on neurological and memory disabilities throughout the population. One of the better-studied brain areas involved in cognitive functions is the hippocampus, which is also an essential brain region targeted by ethanol. Accumulated evidence in several rodent models has shown that ethanol treatment produces cognitive impairment in hippocampal-dependent tasks. These adverse effects may be related to the fact that ethanol impairs the cellular and synaptic plasticity mechanisms, including adverse changes in neuronal morphology, spine architecture, neuronal communication, and finally an increase in neuronal death. There is evidence that the damage that occurs in the different brain structures is varied according to the stage of development during which the subjects are exposed to ethanol, and even much earlier exposure to it would cause damage in the adult stage. Studies on the cellular and cognitive deficiencies produced by alcohol in the brain are needed in order to search for new strategies to reduce alcohol neuronal toxicity and to understand its consequences on memory and cognitive performance with emphasis on the crucial stages of development, including prenatal events to adulthood.

## Introduction

Alcohol is the most common social drug used worldwide, with an average annual consumption of 6.2 L of pure alcohol *per capita* or 13.5 g of pure alcohol per day (WHO, 2014). Alcohol consumption in the population is influenced by different aspects, including the volume of alcohol consumed, the drinking pattern, and the age and gender of the drinker (Sloan et al., 2011; WHO, 2014; Chaiyasong et al., 2018).

Alcohol impacts the health of consumers in many ways, but the central nervous system is especially affected by alcohol toxicity. In numbers, 4% of the total deaths attributable to alcohol are related to the occurrence of neuropsychiatric disorders such as epilepsy, unipolar depressive disorder, vascular dementia, and Alzheimer’s disease (Shield et al., 2013), and more importantly, 24.6% of the total burden of disease attributable to alcohol is related to neuropsychiatric disorders (WHO, 2014). During pregnancy, alcohol consumption drives to the incidence of fetal alcohol syndrome (FAS), a medical condition wherein children born from alcohol-drinking mothers present learning and memory deficits as well as problems with daily life skills, communication, and socialization (Koob and Le Moal, 2005; Merrill and Carey, 2016). Excessive alcohol consumption among adults produces brain abnormalities, including a clinical syndrome known as alcohol-related dementia (ARD), which is the most common cause of dementia in people younger than 65 years old (Harvey et al., 2003). ARD is poorly diagnosed and difficult to recognize because of the lack of a typical pathophysiological profile in people who suffer from it, and it is different from the Wernicke–Korsakoff syndrome, wherein thiamine deficiency explains the brain abnormalities (Moriyama et al., 2006; Ridley et al., 2013).

Alcohol affects several brain areas such as the prefrontal cortex, the corpus callosum, the cerebellum, and the hippocampus. Substantial evidence suggests that one of the main targets of alcohol toxicity in the brain is the hippocampus; indeed the alcoholic population shows neuronal loss and a reduction in total hippocampal volume as shown by magnetic resonance imaging (Jernigan et al., 1991; Harper, 1998).

The hippocampus is a structure located under the cerebral cortex in the limbic system. It has a unique horseshoe-like shape and contains two regions, the cornu ammonis (CA) and the dentate gyrus (DG). The CA is further divided into four zones, namely, CA1, CA2, CA3, and CA4, all of them principally containing pyramidal cells. The connectivity of these zones is especially depicted in a trilaminar loop, wherein afferences *via* the axons of the entorhinal cortex project into the DG. The granule cells in the DG project mossy fibers onto the dendrites of the CA3 pyramidal neurons, and the axons from the CA3 connect to the CA1 neurons in a so-called Schaffer collateral pathway. From there, signals leave the hippocampus to return to the respective sensory cortices.

The hippocampus is one of the most-studied brain structures and is involved in complex processes such as learning and memory, including recognition memory and spatial processing/navigation (Bird and Burgess, 2008; Stella et al., 2012). Evidence shows that the dorsal (posterior in human) hippocampus develops this function, and damaging this portion strongly impairs the acquisition of learning and memory tasks (Moser et al., 1995; Pothuizen et al., 2004). About spatial processing in the human and rodent brain, the hippocampus works beside the thalamus and cortical areas in the creation of a global positioning system through specialized cells called place cells (Bird and Burgess, 2008). Additionally, the hippocampus is involved in emotional behavior (Toyoda et al., 2011). Particularly, the hippocampus participates in the regulation of emotions by responding to positive emotional pictures or stimuli, including memories of past good moments (Santangelo et al., 2018), *via* connections with the amygdala (Guzmán-Vélez et al., 2016). These emotional aspects of hippocampal function are governed by the ventral hippocampus (Moser and Moser, 1998; Fanselow and Dong, 2010) which, working with the amygdala, mediates the response of the rodent in the fear conditioning paradigm (Anagnostaras et al., 2002).

All of these complex processes are related to changes in the strength of the response of the hippocampal circuits, which include interconnections of the CA3 pyramidal neurons with the CA1 region and the DG, representing an extensive region of excitatory glutamatergic synapses (Rebola et al., 2017). These changes in synaptic strength may involve the different forms of calcium-dependent synaptic plasticity known as long-term potentiation (LTP) and long-term depression (LTD), both of them being strongly related to the cognition processes (Stuchlik, 2014).

Studies in rodents have revealed different hippocampal alterations after alcohol administration (Tarelo-Acuña et al., 2000; Obernier et al., 2002; Morris et al., 2010; Zhao et al., 2013), generating a significant amount of evidence that supports the pathological features found in human brains. However, alcohol’s effects on the hippocampal formation are dependent on the developmental stage, triggering different alterations during gestation, adolescence, and adulthood. In this work, we review published data, from early studies to current evidence, on alcohol’s effects on the structure and the function of the hippocampus, including cognitive abilities, cell number, neuron architecture, and electrical properties and function, with special attention on the function closely related to the excitatory glutamatergic transmission. Considering gestational alcohol exposure through consumption or treatment in adolescent and adult rodents, cumulative evidence across decades increases our understanding of the fetal alcohol spectrum disorders (FASD) and the consequences of alcohol intake and abuse in the human population.

## Hippocampal Effects of Alcohol in Prenatal and Neonatal Development Into Adulthood

In humans, alcohol consumption during pregnancy leads to FAS with effects on the memory and the learning abilities after birth, besides resulting in a very characteristic phenotype (Koob and Le Moal, 2005; WHO, 2014; Merrill and Carey, 2016). FAS is the most common form of cognitive disabilities not related to heredity, and it is part of a wide spectrum of disorders (FASD) caused by alcohol consumption during pregnancy. Besides brain structure abnormalities, children with FAS or FASD show impaired cognition and intellectual abilities and have deficient self-regulation and adaptive skills. Indeed these children are context-specific learners, i.e., they learn information in one context, and they cannot apply this knowledge in another context (Denny et al., 2017; Wilhoit et al., 2017).

Rodent models have been a widely used strategy to study the hippocampal defects produced by alcohol consumption during pregnancy. At the behavioral level, the alcohol treatment of pregnant rats during embryonic days 6–20 (E6–E20) increased the escape latency of pups in the Morris water maze (MWM), measured at postnatal day 22 (P22; Blanchard et al., 1987). MWM is a widely used cognitive task to assess hippocampal performance. The rodents search a hidden platform in a pool using visual cues in the room, and they must learn the location of the platform. Escape latency is the time it takes them to find the platform and escape from the water (Morris, 1984). Cognitive impairment by prenatal ethanol exposure in MWM is persistent in the older pups, P40, P60, and P90 (Gianoulakis, 1990), even when using modified MWM in both males (Matthews and Simson, 1998) and females (An and Zhang, 2013, 2015). However, in a model of ethanol exposure by vapor inhalation in a schedule of 6.5 h of exposition per day between E9 and E20, the adult offspring (P63) did not show a cognitive decline (Oshiro et al., 2014). Performance in the other cognitive tasks has shown alterations in avoidance learning (learning of behavior to avoid a stressful or unpleasant situation) and not in working memory (holding information for processing during short periods; Bond and DiGiusto, 1978). In the object–place paired-associate task, which evaluates spatial processing depending on the medial temporal lobe (hippocampal formation), gestational ethanol exposure impaired the performance of adult rats, which are unable to discriminate between two objects on the basis of their location in the field (Sanchez et al., 2019).

Brain development in rodents and humans differ in the highest velocity of brain growth. In rodents, the highest velocity occurs postnatally, in contrast to humans where this phenomenon occurs in the third trimester of development (Cudd, 2005). With this consideration, a single injection of alcohol in pregnant rats (E8) did not produce effects on spatial memory in the adult offspring, while a single alcohol injection during the postnatal period on P7 produced effects on spatial memory when measured at P98 (Sadrian et al., 2014), indicating a role of the developmental stage on effects of alcohol on hippocampal function (Lee et al., 2016; Breit et al., 2019). Vapor ethanol exposure in the perinatal period (P1–P8) impaired the performance of 5-month-old litters in a MWM probe trial, correlating with deficiencies in the gene expression of glutamatergic synapse proteins in the hippocampus (Zink et al., 2011). Accordingly, two intraperitoneal injections on E8 have indicated an impaired performance in the acquisition phase of MWM in the adolescent female mice, while the adolescent male mice did not show differences in performance. This procedure also showed differences in the shape of the brain regions, including the cerebellum and the hypothalamus, but not in the hippocampus, possibly explaining the mild effects of alcohol exposure on learning and memory at this developmental stage (Fish et al., 2016). Aside from that, the mild defect on MWM performance found in adolescent offspring was not observed in adulthood, indicating that alcohol exposure on E8 did not profoundly affect the hippocampal function (Fish et al., 2018). Further, the administration of two hypodermal injections of alcohol at P7 impaired the performance on the MWM in the training and probe trial of 11-week-old mice, which was a session without the platform (Lee et al., 2016). However, a disadvantage of this model in studying the effects of alcohol in brain development is that it is assumed that the placenta and the mother’s alcohol metabolism do not have a relationship with the effects of alcohol in the fetus. It is also important to consider that, in pups injected at P7, the blood alcohol concentration was approximately 0.5 mg/dl, while in the dams which were also injected, the blood alcohol concentration was similar (0.5–0.6 mg/dl), and it is assumed that the fetus reached the same concentration (Sadrian et al., 2014). In a variant of contextual fear conditioning where the hippocampus and the medial prefrontal cortex are required in proper functioning, neonatal exposure to ethanol impaired the performance of the mice in the task, which could be explained by the alterations in the gene expression of the medial prefrontal cortex and not the hippocampus (Heroux et al., 2019), suggesting that the cognitive disabilities after ethanol exposure could not be entirely related to the hippocampal structure and function.

Chronic alcohol consumption during pregnancy permanently decreases the number of CA1 pyramidal neurons in the offspring of rats, with no changes in the DG granule cells (Barnes and Walker, 1981; González-Burgos et al., 2006), likewise with four intraperitoneal (i.p.) injections of ethanol on E7 (Diaz Perez et al., 1991). Furthermore, the DG had shown a reduction in cell number only after postnatal alcohol administration and at high blood alcohol concentrations (peak of 231 ± 32 mg/dl), suggesting that the CA1 pyramidal cells are more susceptible to alcohol damage than the granule cells in the DG (Miller, 1995). In contrast, the *ad libitum* consumption of alcohol did not reduce the CA1 cell number in adult rats after gestational alcohol intake (Lobaugh et al., 1991), probably because the rats do not reach greater blood alcohol concentration by *ad libitum* intake. When a binge drinking pattern of alcohol consumption is used on pregnant rats or newborn pups, there is a reduction in the cell density and number in the CA1, CA3, and DG regions only when alcohol is administered in the period equivalent to the last trimester in humans (P4 to P9 in rats) or all three trimesters (E1 to P9; Livy et al., 2003), and there are no significant effects on the cell number when alcohol is administered to the rats in the period equivalent to the first two trimesters (Maier and West, 2001), suggesting that the third trimester equivalent is the developmental period when the hippocampus is more susceptible to the effects of alcohol. Moreover, ethanol exposure during this developmental stage reduces the number of GABAergic interneurons in adulthood (P90), possibly contributing to cognitive impairment (Bird et al., 2018). Binge-like alcohol administration during the gestation period produces neurodegeneration through an apoptotic mechanism (Ikonomidou et al., 2000), which has also been observed by assessing neuronal death in rat primary hippocampal neurons obtained from fetuses exposed to alcohol during the gestation period (Akbar et al., 2006). Neuronal loss seems to depend on the blood alcohol concentration reached, even if the dose is small. That is, at a lower blood alcohol concentration, the neuronal loss is also lower, and a high blood alcohol concentration produces a higher neuronal loss (Bonthius and West, 1990). On the other hand, the different areas of the hippocampus have shown different vulnerabilities to binge alcohol treatment, with the CA1 and CA4 regions being more vulnerable than the neurons in the CA3 or DG regions (West et al., 1986; Bonthius and West, 1990).

By analyzing neurogenesis in DG neurons after pre- and postnatal alcohol consumption, researchers have found interesting effects regarding 5-bromo-2′-deoxyuridine (BrdU, a marker of cell in active proliferation)-positive cells. The consumption of alcohol in pregnant female rats during the gestational period (E1–E21) did not produce a change in the number of BrdU-positive cells when litters were measured at 6 days old. However, there was a reduction in the number of cells co-labeling with BrdU and NeuN or BrdU and GFAP (Uban et al., 2010). When ethanol consumption was given in the third-trimester-equivalent in humans (P4–P9 in rats), there were less BrdU-positive cells in the 80-day-old rats. Upon calculating an estimate of new NeuN-positive cells per unit volume using BrdU-positive cells per unit volume, the volume density of double-labeled neurons was found to be decreased in the ethanol-treated animals analyzed at 50 and 80 days old (Klintsova et al., 2007). Using the 7-day-old CD-1 mice, a single subcutaneous injection of ethanol reduces the number of BrdU-positive cells in the 5-month-old mice in DG, when BrdU was injected 1 month earlier. The other effects found were reductions in DCX and PCNA immunoreactivity especially in the dorsal hippocampus, reduction in cells co-labeled with Sox2 and GFAP, and increased caspase-3 immunoreactivity in the subgranular zone of DG. All of these findings indicate that a single dose of perinatal ethanol diminishes the progenitor cells and thus reduces adult neurogenesis (Ieraci and Herrera, 2007). Pregnant mice administrated with alcohol for 10 days, starting from gestational day 7, produced a reduction in DCX-positive cells but not Ki-67-positive cells in offspring prenatally exposed to alcohol when analyzed at postnatal day 56, suggesting that prenatal alcohol exposure maintains the damage throughout the adolescent and adult lifespan (Olateju et al., 2018). This observation is also perceived in other species, as macaques prenatally exposed to alcohol showed decreased neurogenesis in the young adult stage (Fedorchak and Miller, 2019).

At the neuronal level, the structure is also altered by ethanol consumption during pregnancy. Prenatal alcohol exposure has shown a reduction in dendrite length in P14 mice (Davies and Smith, 1981), and ethanol exposure on P4–P9 increases the complexity of the apical dendrites in the CA1 pyramidal neurons measured at P9 (Goeke et al., 2018). The dendritic spines are the major structures of excitatory synapses, which receive inputs from the other neurons (Harris and Kater, 1994). The establishment of a mature dendritic spine allows a functional connection between the neurons, while alterations in the number, the density, or the proportion of mature/immature spines have been related to neurological disorders (Fiala et al., 2002). Alcohol exposure of pregnant rats also generates a reduction in the dendritic spine density in the hippocampal pyramidal neurons when pups were tested at P15 after four i.p. injections since the seventh day of gestation (Ferrer et al., 1988) or at P65 after weeks of alcohol treatment (Diaz Perez et al., 1991), and importantly, prenatal alcohol exposure also increases the immature spines and decreases the mature spine numbers at P30–40 (Tarelo-Acuña et al., 2000; González-Burgos et al., 2006). Nevertheless, in some models, at 2 months after birth (~P60), the litters showed a recovery in the mature spines and the spine density (Ferrer et al., 1988; Tarelo-Acuña et al., 2000), which could be related with the differences in the time of exposure and the blood ethanol concentration reached. Two ethanol injections during E8 produce transient changes in the structure of the pyramidal neurons at P1, which are fully recovered at P10, including the dendritic spine density (Jakubowska-Dogru et al., 2017), suggesting the importance of the developmental stage in ethanol exposure and recovery. On the other hand, differences have been reported in the establishment of synapses, with a decrease in the total synapses, the simple synapses, and the symmetric synapses in the molecular layer of the DG at P30 (Hoff, 1988) as well as alterations in the mossy fiber topography at P60 (West et al., 1981). All of these evidences point to the fact that alcohol consumption during gestation alters synapse formation and maturation, and these alterations could remain during adolescence and adulthood.

Finally, the electrical properties and functions have been shown as altered under prenatal alcohol exposure. The litters exposed prenatally to alcohol and evaluated at P50 showed an impaired LTP induction and an epileptic behavior in the CA1 region of the hippocampus (Swartzwelder et al., 1988). The LTP impairment was also observed in the DG *in vivo*, although without changes in the input–output (*I*/*O*) curves (Sutherland et al., 1997; Patten et al., 2013), suggesting an impaired ability to evoke potentiation without changes in the basal synaptic transmission in the hippocampus. Neonatal ethanol exposure also impaired the LTP in neonatal rats only at a high ethanol concentration (300 mg/dl) and without changes in the basal synaptic transmission, suggesting defects in synaptic plasticity (Puglia and Valenzuela, 2010). Gestational alcohol administration impaired the LTP in P36 offspring, and depotentiation was facilitated in the male offspring, while enhanced LTP and suppressed depotentiation were seen in females, suggesting a gender-dependent differential effect (An and Zhang, 2013, 2015). In another independent study, LTP impairment on the DG was observed only in males, while LTP was enhanced in females in a protocol of alcohol intake throughout pregnancy and recorded during adolescence (P30; Titterness and Christie, 2012) or during adulthood (Sickmann et al., 2014) of the offspring. Contrary results in juvenile offspring are also available, showing LTP impairment on the DG in both sexes (Fontaine et al., 2019). Surprisingly, both studies used the same experimental procedure; however, the juvenile rats were assessed on different time points, P30–35 and P21–28, respectively, which could explain the opposite results on females. Further studies are necessary to address the sex differences on LTP after prenatal alcohol exposure. Alcohol exposure at three different times, equivalent to the three human trimesters, and evaluated at adulthood show an impaired dentate LTP more dramatically than when it was administered during the second-trimester-equivalent, supporting the evidence of specific periods of susceptibility to the toxic effects of ethanol during development (Helfer et al., 2012).

*In vivo* recordings of CA1 neurons have identified changes in theta activity, a type of oscillatory pattern in the hippocampus when the rodent is in active motor behavior and exploration. After prenatal alcohol exposure, both an increase in theta activity during movement and a decrease in theta activity during stillness were observed (Cortese et al., 1997). The DG *in vivo* recordings have indicated that prenatal alcohol exposure decreases the excitability of granule cells, as indicated by the reduced correlation between field excitatory postsynaptic potential (fEPSP) and population spikes (PS) amplitude, and impairs the maintenance of LTP once evoked (Varaschin et al., 2014), confirming the observations obtained in *ex vivo* brain slices for CA1 and DG LTP.

In summary, alcohol consumption during pregnancy alters the development and function of the hippocampus. Hippocampal impairment persists during adolescence and adulthood in the alcohol-treated dam’s litters. This hippocampal impairment could probably be due to a decrease in the number of neurons, especially in the CA1 (a cell population more vulnerable to alcohol), an altered dendritic structure, and a reduced number of synapses, which also correlate with the alterations in the electrical behavior of neurons unable to potentiate in response to the high-frequency stimulations.

## Alcohol Consumption in Young Rodents

Brain development persists during childhood and adolescence in mammals. Alcohol consumption is not only risky during the pre-natal stages, but adolescence is also a crucial period in the maturation of the brain and its circuitry, where alcohol toxicity could cause damage in a long-lasting way. Furthermore, adolescence is usually the age for the start of alcohol consumption and abuse in humans; so, it is important to know the consequences that this behavior can have.

Comparing adult and young male rats, alcohol treatment at 30 min before MWM testing impaired the rats’ performance in the acquisition phase and during the probe trial of the MWM task only in the young animals (Markwiese et al., 1998), suggesting that adolescents are more susceptible to alcohol-induced hippocampal dysfunction than the adults. In another study, acute administration caused poor cognitive performance in MWM in both adolescent and adult rats. Nevertheless, hippocampal dysfunction lasts up to 25 days later in adolescent rats, in contrast to adults where re-testing did not show differences between saline- and ethanol-treated rats (Sircar and Sircar, 2005). In the same line of evidence, female young and adult rats showed an impaired cognitive performance in the acquisition phase of MWM, while only young rats displayed a poor cognitive performance during the probe trial (Sircar et al., 2009), supporting the hypothesis that the adolescent and young populations are more vulnerable to alcohol-induced hippocampal dysfunction.

The binge drinking pattern of consumption becomes important especially in the adolescent population (Kuntsche et al., 2005). This pattern of alcohol consumption leads to high blood alcohol concentrations (>80 mg/dl) in a very short time (NIAAA), as what occurs in typical teenage parties. The binge-like protocols trigger adverse effects on the central nervous system function and can produce significant consequences over time (Merrill and Carey, 2016; Tapia-Rojas et al., 2017). The binge-like alcohol treatment induced an impairment of spatial processing and recognition memory even at 1 week later from the binge episode, while cognitive performance is re-established at 10 weeks later from the binge episode (Silvestre de Ferron et al., 2016; Tapia-Rojas et al., 2018) or as soon as 14 days from ethanol withdrawal in a binge drinking model in female rats (Fernandes et al., 2018). The re-establishment of recognition memory performance has been recorded as soon as 3 weeks after the last alcohol exposure in a binge-like paradigm (Tapia-Rojas et al., 2018). Contrary results have been published upon using vapor chambers compared to binge alcohol exposure. Alcohol administration for 10 h per 3 days for 4 weeks revealed the absence of a cognitive impairment in the MWM acquisition or probe trials (Schulteis et al., 2008), as well as treatment for 16 h per day for 4 days (Van Skike et al., 2012). However, the working memory was effectively impaired using this methodology in the MWM (Schulteis et al., 2008), indicating no alterations in spatial processing that depend on the hippocampus, which is contrary to the observations under the other binge-like administration protocols cited before. As we previously mentioned on prenatal alcohol treatment, alcohol vapor inhalation did not induce a hippocampal impairment in cognitive tasks, raising the possibility that in some way opposite results can be related to the differences in the way of administration, the time needed to reach high alcohol concentrations in the blood, and the actual blood concentration reached.

In the same line, binge alcohol treatment in young rats decreased neurogenesis, and new cells died probably by necrosis, given the low percentage of TUNEL-positive cells compared with pyknotic nuclei, wich is a characteristic of dead cells (Morris et al., 2010). When the DCX marker was studied, a decreased DCX immunoreactivity was observed after 4 days of binge ethanol treatment and after 2 days of withdrawal, but not after 7 days of withdrawal. After 28 days of withdrawal, a reduction in BrdU-positive cells was observed, indicating a reduction in cell survival after ethanol exposure in the adolescent rats (Morris et al., 2010). Nevertheless, intraperitoneal injections of alcohol for 3 days increase the number of TUNEL-positive cells and the number of pyknotic cells (Jang et al., 2002), suggesting neuronal death through an apoptotic mechanism. These pieces of evidence suggest that adolescent alcohol treatment seems to induce neuronal death; however, whether the mechanism is apoptosis or necrosis has not yet been elucidated. McClain also showed the increase in BrdU immunoreactivity not only in the DG but in the hippocampus as well, where colocalization with Iba1 was strongly observed in the adolescent rats (McClain et al., 2011). In the other studies using adolescent rats, the number of BrdU-positive cells was only higher than the control after 7 days of withdrawal in a 4-day binge protocol, and this result was confirmed with Ki67 immunoreactivity (McClain et al., 2014). Also, in this study, they found that the majority of newborn cells (NeuN-positive as well) were ectopically ubicated; therefore, the new neurons could not integrate properly in the hippocampal circuitry (McClain et al., 2014). Upon evaluating neurogenesis with another alcohol consumption paradigm, a liquid diet *ad libitum* for 2 weeks, there was a reduction in BrdU-positive cells in both males and females (Anderson et al., 2012). In rats, ethanol consumption every 48 h for 20 days (11 exposures at the end) produced a reduction in the DCX immunoreactivity 22 days after the last dose of ethanol in adolescent rats but not in adult rats. This pattern of alcohol intake produced no changes in the Ki67 immunoreactivity in adolescent rats and an increase in the cleaved caspase-3 immunoreactivity, suggesting a persistent loss of neurogenesis only in adolescents and not in adults under the same ethanol protocol (Broadwater et al., 2014).

At the structural level, binge-like treatment in rats produced an increase in the immature spine number with a concomitant reduction in the mature spine number in the CA1 pyramidal neurons (Risher et al., 2015), suggesting a reduction in the number of spines as well as decreasing maturation, which correlates with the poorest excitatory transmission in the hippocampus. Accordingly, the granule neurons in the dorsal hippocampus have shown a reduction in dendritic spine density in the adult rats submitted to intermittent ethanol exposure during adolescence. Concomitantly, a reduction in the mushroom dendritic spines, as well as in the long dendritic spines, was observed in these rats (Mulholland et al., 2018), indicating that adolescent ethanol exposure alters the structure of the dendritic terminals persistently to adulthood.

Concerning the electrical function of the neurons, in mature rats only high concentrations of alcohol (100 mM) produced a decrease in the population excitatory postsynaptic potentials (pEPSPs), while in the immature rats pEPSP changed in a dose-dependent manner, with a significant effect even at 10 mM alcohol (Swartzwelder et al., 1995b). Importantly, the effect of alcohol on pEPSPs that depends on the activity of the ionotropic glutamate receptor *N*-methyl-D-aspartate receptor (NMDAR), a major contributor to LTP and LTD, is not mediated by the subunit GluN2B, a subunit particularly sensitive to regulation (Swartzwelder et al., 1995a). Moreover, using adolescent brain slices (P21–P26), Mameli et al. (2005) showed that the currents through NMDAR are inhibited by alcohol in a dose-dependent manner (10–50 mM), but in neonate brain slices the NMDAR currents were inhibited only at a concentration of 75 mM. The subunit composition of the NMDAR changes during development, and probably this different composition of the NMDAR subunits could explain the way that the hippocampal circuit responds to alcohol at both stages. More recent studies, however, have shown that GluN2B in the extrasynaptic membranes regulates a greater number of proteins than GluN2B in the synaptic membranes in adult rats submitted to intermittent ethanol exposure during adolescence (Swartzwelder et al., 2016). In mice, the chronic intermittent ethanol exposure alters GluN2B interaction with proteins important for a mechanism of LTD dependent on the metabotropic glutamate receptors (Wills et al., 2017), raising a new possible mechanism for cognitive impairment provoked by adolescent alcohol consumption.

The alcohol-related effects are suggested to be dependent on how alcohol concentration is increased. The CA1 recordings of fEPSPs showed that 60 mM alcohol produced a blockage of LTP induction, but when alcohol was administered in a stepwise manner in 10-mM increments every 15 min until reaching 60 mM, there was no blockage of the LTP, possibly indicating tolerance. This tolerance seems to be related to the alterations in the intracellular calcium storages and/or metabotropic glutamate receptor function (Tokuda et al., 2007), adding a new step of complexity in the effects of alcohol on hippocampal function.

The *ex vivo* slices exposed to alcohol showed alterations in synaptic plasticity in the other regions of the hippocampus, such as the perforant path. In the DG, the application of 75 mM alcohol produced an inhibition of LTP and an inhibition of hyperexcitability after a paired pulse, inhibiting NMDAR signaling more than affecting the inhibitory gamma-amino butyric acid receptors (Morrisett and Swartzwelder, 1993), in contrast to presynaptic GABA-A receptors which are very sensible to ethanol in the hippocampus and regulate glutamate release (Wakita et al., 2012). When the CA3 neurons were analyzed as the postsynaptic compartment (stimulating the perforant path), 50 mM alcohol produced an important decrease in the amplitude of another ionotropic glutamate receptor, the α-amino-3-hydroxy-5-methyl-4-isoxasolpropionic-acid-mediated excitatory postsynaptic currents in neonatal slices, but not in young rats (Mameli et al., 2005), suggesting the differing effects of alcohol in the glutamatergic neurotransmission in the hippocampus during development. Paired pulse facilitation (PPF) showed a decrease in glutamate release in the CA3 region of the neonate rats, but not in the juvenile rats (Mameli et al., 2005), and in the CA1 region (Hendricson et al., 2004), which could probably be due to the action of alcohol on the N-type calcium channels (voltage-gated calcium channels; Mameli et al., 2005), altering the amount of glutamate released by an action potential. There are forms of LTP independent of the activity of the NMDAR, which are also inhibited by the alcohol and GABAergic transmission, which modulates this type of plasticity, mediating the inhibitory effect of alcohol at least in part (Izumi et al., 2005).

The animals treated with alcohol in a vapor chamber model showed that the neurotransmitter release is altered after 1 day of withdrawal, but not after 7 days of withdrawal as revealed by PPF. On the contrary, the synaptic strength is reduced after 7 days of withdrawal and not after 1 day of withdrawal as revealed in the *I*/*O* curves (Nelson et al., 2005), probably suggesting a time relationship between the presynaptic and the postsynaptic effects. Following a schedule of 2-days-on and 2-days-off intraperitoneal injections of alcohol for 2 weeks (binge-like pattern), alcohol provoked a decrease in the slope of the *I*/*O* curves at 1 week after the protocol ended and an impairment of PPF, indicating the pre- and postsynaptic effects of the treatment, which are compensated and re-established to control conditions 3 or 7 weeks after the protocol (Tapia-Rojas et al., 2018). The effect of only two episodes of alcohol intoxication has been studied, and two injections of alcohol at toxic concentrations abolished the LTD at 48 h after the protocol ended. A possible mechanism is a change in the composition of the NMDAR subunits, indicating a shift toward increased GluN2B subunit expression (Silvestre de Ferron et al., 2016; Drissi et al., 2019).

Alcohol administration in a binge-like protocol during adolescence changes the action of alcohol on the GABAergic transmission in the adult DG. Specifically, the adolescent alcohol treatment makes the extrasynaptic GABA-A receptors more vulnerable to alcohol in adulthood and makes the synaptic GABA-A receptors less sensitive to alcohol in adulthood (Fleming et al., 2012), indicating that adolescent alcohol exposure drives long-lasting changes in the GABA-A receptors and the DG. Importantly, this effect is specific to a binge-like alcohol treatment during adolescence, while the same results were not found in the rats treated during young adulthood or adulthood (Fleming et al., 2013). Additionally, this binge drinking pattern of consumption during adolescence produces long-lasting effects on the essential potassium currents in the inhibitory interneurons of the CA1 region (Li et al., 2013).

## Alcohol Consumption in Adult Rodents

The alcohol-related effects on hippocampal function have been widely studied in adult rodents under different paradigms and experimental designs which are reviewed here.

The administration of alcohol acutely before performing a cognitive test impairs the hippocampal function in adult rodents (2 months and beyond). The mice and rats showed an impaired cognitive performance in the radial arm maze (Gibson, 1985; Matthews et al., 1995; White et al., 1997), the T- maze (Givens, 1995), and the MWM (Shimizu et al., 1998), all of which evaluate spatial memory and thus hippocampal function (Olton and Samuelson, 1976; Morris, 1984; Poucet and Benhamou, 1997; Dubreuil et al., 2003), suggesting the altered neurotransmission in the hippocampus. The fear conditioning test is a behavioral test that evaluates hippocampal function without evaluating spatial memory but assesses the processing of contextual information (Chang and Liang, 2017). It has been reported that the hippocampus is involved in contextual and not tone cue conditioning during this type of test (Kim and Jung, 2006). Acute alcohol administration decreases the freezing time in the contextual conditioning phase of the test, but not in the tone conditioning phase of the test (Melia et al., 1996), revealing an alcohol-triggered hippocampal dysfunction in a task different from spatial memory.

Chronic administration of alcohol did not impair the cognitive performance in MWM treated for 26 or 30 weeks (Blokland et al., 1993; Lukoyanov et al., 2000). However, when the short-term (4 weeks) and long-term (36 weeks) modes of administration were compared, the longer period induced an impaired cognitive performance (Franke et al., 1997), suggesting that periods of consumption longer than 30 weeks are necessary to produce detrimental effects on the hippocampal function. This conclusion does not consider the possible effect of aging on the hippocampal function, and maybe there is a relationship between both processes. In a C57BL/6J mouse model of 3 weeks of chronic free choice, alcohol intake produced an impairment in the conditioning phase and in the context test of the fear conditioning test, but there were no effects on the NOR task nor the Barnes maze task (Stragier et al., 2015), indicating a mild cognitive impairment and hippocampal dysfunction.

Chronic alcohol administration has also been associated with impaired performance in the other cognitive tasks such as the Hebb–Williams maze (working memory assessment; Bond and Digiusto, 1976; Fehr et al., 1976), the radial arm maze (spatial memory; Gál and Bárdos, 1994), the spontaneous alternation paradigm (spatial processing), the attentional set shifting (cognitive flexibility, dependent of frontal cortex; Vedder et al., 2015), the step-down passive avoidance task, the Greek cross maze, and the Shuttlebox task (Farr et al., 2005), all of them of which are related with avoidance learning, which involves the limbic system including the hippocampus (Gabriel, 1993). Chronic intermittent ethanol exposure (CIE) in vapor chambers increases the anxiety-like behaviors in the adult male rats, possibly related to the alterations in the synaptic activity of the ventral hippocampus and not of the dorsal hippocampus (Ewin et al., 2019).

The chronic alcohol consumption in rats induced a reduction in the number of hippocampal pyramidal neurons and granule cells after several months of intake and withdrawal (Walker et al., 1980; Cadete-Leite et al., 1988; Paula-Barbosa et al., 1993; Franke et al., 1997; Lukoyanov et al., 2000). A decreased number of granule cells is evident as soon as after 4 months of alcohol treatment (Lukoyanov et al., 2000) and persists after 5 months of treatment and 2 months of withdrawal (Walker et al., 1980). However, as mentioned previously, cognitive decline is observed when chronic consumption is sustained in time, at over 30 weeks of treatment. Thus, the reduction in the number of granule and pyramidal cells does not necessarily correlate with the cognitive decline, suggesting that compensatory mechanisms are relevant to maintain the cognitive performance. Interestingly, chronic alcohol consumption during 9 months, followed by 3 or 6 months of withdrawal, produces neuronal loss in the hippocampus, although there was a slight effect of aging itself (Lescaudron and Verna, 1985). In a protocol of intermittent ethanol exposure in a 2-days-on and 2-days-off paradigm in rats, with evaluation at 1, 25, or 165 days after the last dose of ethanol, the DCX immunoreactivity decreased in the dorsal and the ventral hippocampus 25 days after the last dose of ethanol (postnatal day 80), the Ki67 immunoreactivity was decreased, and the cleaved caspase-3 immunoreactivity was augmented in both the dorsal and the ventral hippocampus (Vetreno and Crews, 2015). In a vapor chamber model, the Ki67 immunoreactivity was decreased 15 days after the cessation of the protocol, while the DCX was decreased 15 and 56 days after the protocol ended. The ethanol vapor paradigm produced a persistent reduction in the proliferating NPC, which were becoming immature neurons, leading to a severe loss of neurogenesis (Ehlers et al., 2013). Importantly, the loss of neurogenesis also has been documented in post-mortem samples retrieved from an adult human well characterized for alcohol abuse (Dhanabalan et al., 2018; Le Maître et al., 2018), suggesting that adult alcohol abuse has a broad effect among species and therefore more pharmacological testing has to be done in rodents.

Furthermore, 4 months of alcohol intake did not produce a significant reduction in the CA1 neurons, but after 4 months of withdrawal it was possible to observe a reduction in the CA1 pyramidal neurons, indicating that cell loss could be mediated more by the withdrawal period than alcohol consumption itself (Phillips and Cragg, 1983). In mice, chronic alcohol consumption has shown cell death in the DG by an apoptotic mechanism, increasing the cleaved caspase-3 immunoreactivity, and which is decreased using memantine (Wang et al., 2018), an NMDAR inhibitor, suggesting excitotoxic cell death. Binge alcohol drinking in adults has also been associated with the degeneration of neurons. Intermittent intraperitoneal injection of alcohol for 1 month caused a reduction in the number of pyramidal cells in the hippocampal CA3 region (Lundqvist et al., 1995). Also, Fluoro-Jade B staining, which recognizes neurons undergoing degeneration, increased in the granule cells of the DG and the entorhinal cortex, the main afference of the hippocampus, indicating neurotoxicity caused by the binge alcohol treatment (Cippitelli et al., 2010b), and it is prevented using a group II metabotropic glutamate receptor agonist along with a recovery in reversal learning in the MWM task (Cippitelli et al., 2010a). However, the mechanisms underlying the neuronal cell death are not conclusive. Previously, it has been proposed that binge alcohol intake produces neuronal loss by necrosis 2 days after administration (Obernier et al., 2002), while the other reports showed an increase in caspase-3 immunoreactivity, which co-labeled with NeuN (Qin and Crews, 2012), indicating apoptosis. The administration of repeated cycles of the binge-like alcohol treatment provoked an increase in Fluoro-Jade B staining in the hippocampus, which decreased to control values 2 weeks after the last dose of alcohol, indicating a decrease in cell death along with the withdrawal (Zhao et al., 2013). In female rats, binge drinking has been associated with a reduction in the number of cells in the DG despite the fact that neurogenesis is increased in this hippocampal region and there is a lack of cognitive impairment in the MWM (West et al., 2019), suggesting that neuronal loss did not correlate with cognitive decline, and neurogenesis is not sufficient to recover the cell number in the DG. Thus, there is an important difference between chronic and binge alcohol consumption. The first one seems to produce neuronal loss during the withdrawal period, while binge alcohol consumption induces neuronal death because of alcohol consumption itself.

Several studies have documented changes at the synaptic level, like an increase in the dendritic length of the granule cells (Cadete-Leite et al., 1988; Durand et al., 1989; Paula-Barbosa et al., 1993), which returned to control values after a long withdrawal period (Cadete-Leite et al., 1989a; Paula-Barbosa et al., 1993), and an increase in the percentage of the plasmalemma of mossy fibers occupied by synaptic contacts (Cadete-Leite et al., 1989b; Paula-Barbosa et al., 1993). In agreement, an important finding was reported by Lukoyanov et al. (2000) who, despite observing fewer neurons in the hippocampus, found no change in the number of total synapses between the mossy fibers and the CA3 pyramidal neurons, suggesting that, after chronic alcohol consumption, there is a remodeling of synaptic connections that might compensate for the loss of neurons and that can be correlated with the absence of cognitive impairment as discussed earlier. In 1978, Riley and Walker, using a model of chronic alcohol exposure in mice (4 months of consumption and 2 months of withdrawal), observed 50–60% reduction in the dendritic spine number in the dentate granule cells and the CA1 pyramidal neurons in mice (Riley and Walker, 1978). Two years later, a conflicting evidence was reported using chronic alcohol consumption in rats treated for 4 months (Lee et al., 1981) or for 12 months with 6 months of withdrawal (Cadete-Leite et al., 1989a). However, more evidences have emerged, supporting the effects of chronic alcohol exposure on the dendritic spines. Chronic alcohol consumption or administration produced a reduction in the number of the dendritic spines in the posterior and the anterior hippocampus in mice (Lescaudron et al., 1989) and rats (McMullen et al., 1984) and in the CA1 region of the hippocampus (King et al., 1988). Interestingly, in all these cases, alcohol withdrawal led to a recovery in the hippocampus spine number compared to the control values (McMullen et al., 1984; King et al., 1988; Lescaudron et al., 1989). In contrast, in the granule cells of the DG, the treatment increased the dendritic spines, followed by a reduction in spines during the withdrawal period (King et al., 1988). A more recent report, using alcohol treatment in a vapor chamber with a CIE model, showed a reduction in dendritic complexity that persists after abstinence and increases in spine density, which is reduced after prolonged abstinence in the DG. In contrast, the CA1 and CA3 cells showed more dendritic arborization during CIE, followed by a reduction comparable to that in controls after abstinence. Spine density in the CA3 and the CA1 did not change after CIE, but there was a reduction in the density after abstinence. In spite of the lack of correlation between the architectural changes and the dendritic spine density, the NMDAR subunit composition changes more accurately with architecture, increasing after CIE and decreasing after withdrawal (Staples et al., 2015). The dendritic spine density decreases in rats after four cycles of binge alcohol treatment and three cycles of withdrawal. However, at 14 days after the last dose of alcohol, there was a recovery in the dendritic spine density in the hippocampus and the entorhinal cortex (Zhao et al., 2013). To date, there is limited information about the effects of binge drinking on the synaptic connections or the dendritic spines.

Electrical recordings in *ex vivo* brain slices from 150-g to 200-g rats (approximately 6 weeks old) have indicated that 100 mM alcohol in the recording solution decreases the magnitude of LTP in the CA3–CA1 hippocampal synapse, while 50 mM alcohol did not change the LTP evocation (Sinclair and Lo, 1986), indicating that the inhibition of potentiation is dose dependent. Also, the process is reversible because when alcohol is washed out, the LTP is recovered, achieving control values after a second stimulation (Blitzer et al., 1990). In the CA1 hippocampal field, 20 mM alcohol enhanced the inhibitory postsynaptic currents mediated by the GABA-A receptors, and this enhancement depends on the phosphorylation state of the receptor (Weiner et al., 1994).

The consumption of an aqueous solution of alcohol for 18 days prevented the induction of LTP in rat *ex vivo* slices (Johnsen-Soriano et al., 2007). The LTP impairment appears potentiated by the repeated withdrawals, not by a single withdrawal period, without changes in the *I*/*O* curves, in an oral model of alcohol consumption in rats (Stephens et al., 2005). Using mice, free-choice alcohol consumption for 21 days did not provoke LTP impairment in the LTP induced by 5 or 10 TBS bursts (Stragier et al., 2015).

Chronic alcohol consumption experiments using the CIE paradigm in a vapor chamber (12–14 days) have revealed a decrease in post-tetanic potentiation (PTP) and even the absence of LTP in the CA1 region. The PPF showed alterations only during PTP, indicating a reduced release of neurotransmitters. All of these findings were still present 1 day after withdrawal; however, at 5 days after withdrawal, at least the dendritic component of LTP showed recovery compared to the control group (Roberto et al., 2002). In agreement, the microdialysis experiments have shown that basal glutamate concentration is increased after 6 days of moderate doses of ethanol, indicating an increased neurotransmitter release under basal conditions (Chefer et al., 2011). This increase in extracellular glutamate in basal conditions could explain the alterations in the PPF. An increase in the basal glutamate release could reduce the number of exocytosis vesicles primed at the plasma membrane, explaining why a second pulse close enough is not sufficient to release more glutamate when the presynaptic calcium concentration is high.

Electrophysiological experiments performed *in vivo*, with the use of stereotaxic surgeries, have shown that alcohol i.p. administration acutely produced a decrease in the PS of the DG and CA1 neurons without effects on the fEPSPs. The firing rate was also decreased in both the DG and CA1 neurons, with no effects on interneurons (Steffensen and Henriksen, 1992), and LTP induction was altered in the DG (Steffensen et al., 1993). LTP is also inhibited *in vivo* by systemic alcohol administration at doses of 0.5 and 1.0 g/kg (Givens and McMahon, 1995). A 5% alcohol solution perfused intrahippocampally on the CA1 region produced a decrease in the neuronal firing and a slow-wave sleep pattern (Ludvig et al., 1995). Alcohol also decreased the NMDA-evoked activity in the hippocampal recordings *in vivo*; however, alcohol did not decrease the glutamate-evoked activity (Simson et al., 1993). A recording of hippocampal place cells during the radial arm maze procedure showed that a single alcohol injection (2 g/kg) 30 min before the recordings altered the place specificity of firing. The place specificity recovered after a withdrawal period of at least 24 h (Matthews et al., 1996).

## Conclusions

Despite the discovery of the negative impact of alcohol on health, alcohol consumption persists at high rates worldwide. Hippocampal function in the temporal lobes has been described as an alcohol target, and rodent models have allowed understanding its effects.

**Figure 1:**
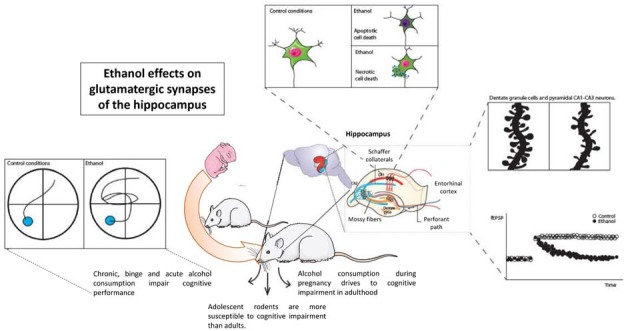
Ethanol effects on hippocampal synaptic transmission and hippocampal function. Considering wide evidence, ethanol acutely alters the function of the hippocampus as measured in different cognitive tasks. A large amount of evidence shows alterations in structural plasticity inside hippocampal neurons, either dentate granule cells or pyramidal neurons. Glutamatergic transmission, especially that involving NMDAR function such as long-term potentiation, is also altered by ethanol.

Alcohol alters the cognitive abilities in rodent models. Alcohol administered acutely, chronically, or in a binge-like pattern severely impairs hippocampal function and cognitive performance in all stages of development. Prenatal alcohol exposure impairs the hippocampal functions, which lasts long into adolescence and adulthood, while adolescent alcohol exposure is more harmful to the cognitive abilities than adult alcohol exposure is, establishing that the developing brain is more vulnerable to the toxic effects of alcohol. Alcohol consumption triggers neuronal death in the hippocampal zones either by a necrotic or apoptotic mechanism, but conclusive results do not presently exist. The neurons alter their connections, including a decrease in the dendritic spine density and remodeling their synaptic contacts to maintain the number of connections between the neurons in response to chronic or binge alcohol patterns. Alcohol exerts its effects on the glutamatergic transmission by altering the NMDAR function and kinetics, while fewer variations in other fast glutamatergic currents have been reported. The structural changes observed are in concordance with the detrimental effects of alcohol on the LTP and the LTD, as well as other electrical measures of the glutamatergic transmission and the GABAergic transmission (Figure 1).

The evidence of the harmful effects of alcohol on the hippocampal formation is wide, and the structural and functional effects are understood. There is still considerable research needed to elucidate how short- or medium-term alcohol exposure can affect the long-term hippocampal performance and how they can contribute to the development of ARD. The complexity of alcohol research and its translation to human consumption falls on the number of variables to consider, such as age, pattern and time of ingesting, volume consumed, and physiological state of the subjects. For this reason, it is important to evaluate and introduce different models that can provide insight into how alcohol alters the cognitive processes in the brain and how it can perturb the cognitive state of consumers.

## Author Contributions

RM and WC: conception of the idea. RM: bibliographic research and main drafting. ML, CT-R, DR, and RQ: drafting and revising the article. WC: revising the article and correspondence.

## Conflict of Interest

The authors declare that the research was conducted in the absence of any commercial or financial relationships that could be construed as a potential conflict of interest.

## References

[B1] Akbar M. Baick J. Calderon F. Wen Z. Kim H. Y. (2006). Ethanol promotes neuronal apoptosis by inhibiting phosphatidylserine accumulation. J. Neurosci. Res. 83, 432–440. 10.1002/jnr.20744 16397898

[B2] An L. Zhang T. (2013). Spatial cognition and sexually dimorphic synaptic plasticity balance impairment in rats with chronic prenatal ethanol exposure. Behav. Brain Res. 256, 564–574. 10.1016/j.bbr.2013.09.017 24050890

[B3] An L. Zhang T. (2015). Prenatal ethanol exposure impairs spatial cognition and synaptic plasticity in female rats. Alcohol 49, 581–588. 10.1016/j.alcohol.2015.05.004 26251263

[B4] Anagnostaras S. G. Gale G. D. Fanselow M. S. (2002). The hippocampus and Pavlovian fear conditioning: reply to Bast et al. Hippocampus 12, 561–565. 10.1002/hipo.10071 12201641

[B5] Anderson M. L. Nokia M. S. Govindaraju K. P. Shors T. J. (2012). Moderate drinking? Alcohol consumption significantly decreases neurogenesis in the adult hippocampus. Neuroscience 224, 202–209. 10.1016/j.neuroscience.2012.08.018 22906480 PMC4568748

[B6] Barnes D. E. Walker D. W. (1981). Prenatal ethanol exposure permanently reduces the number of pyramidal neurons in rat hippocampus. Brain Res. 227, 333–340. 10.1016/0165-3806(81)90071-7 7260643

[B7] Bird C. M. Burgess N. (2008). The hippocampus and memory: insights from spatial processing. Nat. Rev. Neurosci. 9, 182–194. 10.1038/nrn2335 18270514

[B8] Bird C. W. Taylor D. H. Pinkowski N. J. Chavez G. J. Valenzuela C. F. (2018). Long-term reductions in the population of GABAergic interneurons in the mouse hippocampus following developmental ethanol exposure. Neuroscience 383, 60–73. 10.1016/j.neuroscience.2018.05.003 29753864 PMC5994377

[B9] Blanchard B. A. Riley E. P. Hannigan J. H. (1987). Deficits on a spatial navigation task following prenatal exposure to ethanol. Neurotoxicol. Teratol. 9, 253–258. 10.1016/0892-0362(87)90010-9 3627089

[B10] Blitzer R. D. Gil O. Landau E. M. (1990). Long-term potentiation in rat hippocampus is inhibited by low concentrations of ethanol. Brain Res. 537, 203–208. 10.1016/0006-8993(90)90359-j 2150775

[B11] Blokland A. Prickaerts J. Raaijmakers W. (1993). Absence of impairments in spatial and temporal discrimination-learning in Lewis rats after chronic ethanol-consumption. Pharmacol. Biochem. Behav. 46, 27–34. 10.1016/0091-3057(93)90312-h 8255920

[B12] Bond N. W. Digiusto E. L. (1976). Impairment of Hebb-Williams maze performance following prolonged alcohol consumption in rats. Pharmacol. Biochem. Behav. 5, 85–86. 10.1016/0091-3057(76)90292-6 996044

[B13] Bond N. W. DiGiusto E. L. (1978). Avoidance conditioning and Hebb-Williams maze performance in rats treated prenatally with alcohol. Psychopharmacology 58, 69–71. 10.1007/bf00426792 97722

[B14] Bonthius D. J. West J. R. (1990). Alcohol-induced neuronal loss in developing rats: increased brain damage with binge exposure. Alcohol. Clin. Exp. Res. 14, 107–118. 10.1111/j.1530-0277.1990.tb00455.x 1689970

[B15] Breit K. R. Zamudio B. Thomas J. D. (2019). The effects of alcohol and cannabinoid exposure during the brain growth spurt on behavioral development in rats. Birth Defects Res. 111, 760–774. 10.1002/bdr2.1487 30854806 PMC7749758

[B16] Broadwater M. A. Liu W. Crews F. T. Spear L. P. (2014). Persistent loss of hippocampal neurogenesis and increased cell death following adolescent, but not adult, chronic ethanol exposure. Dev. Neurosci. 36, 297–305. 10.1159/000362874 24993092 PMC4125431

[B17] Cadete-Leite A. Tavares M. A. Alves M. C. Uylings H. B. Paula-Barbosa M. M. (1989a). Metric analysis of hippocampal granule cell dendritic trees after alcohol withdrawal in rats. Alcohol. Clin. Exp. Res. 13, 837–840. 10.1111/j.1530-0277.1989.tb00433.x 2690669

[B18] Cadete-Leite A. Tavares M. A. Pacheco M. M. Volk B. Paula-Barbosa M. M. (1989b). Hippocampal mossy fiber-CA3 synapses after chronic alcohol consumption and withdrawal. Alcohol 6, 303–310. 10.1016/0741-8329(89)90087-6 2765199

[B19] Cadete-Leite A. Tavares M. A. Uylings H. B. Paula-Barbosa M. (1988). Granule cell loss and dendritic regrowth in the hippocampal dentate gyrus of the rat after chronic alcohol consumption. Brain Res. 473, 1–14. 10.1016/0006-8993(88)90309-5 3208112

[B20] Chaiyasong S. Huckle T. Mackintosh A. M. Meier P. Parry C. D. H. Callinan S.. (2018). Drinking patterns vary by gender, age and country-level income: cross-country analysis of the International Alcohol Control Study. Drug Alcohol Rev. 37, S53–S62. 10.1111/dar.12820 29900623 PMC6120521

[B21] Chang S. D. Liang K. C. (2017). The hippocampus integrates context and shock into a configural memory in contextual fear conditioning. Hippocampus 27, 145–155. 10.1002/hipo.22679 27806432

[B22] Chefer V. Meis J. Wang G. Kuzmin A. Bakalkin G. Shippenberg T. (2011). Repeated exposure to moderate doses of ethanol augments hippocampal glutamate neurotransmission by increasing release. Addict. Biol. 16, 229–237. 10.1111/j.1369-1600.2010.00272.x 21182572 PMC3684957

[B23] Cippitelli A. Damadzic R. Frankola K. Goldstein A. Thorsell A. Singley E.. (2010a). Alcohol-induced neurodegeneration, suppression of transforming growth factor-β, and cognitive impairment in rats: prevention by group II metabotropic glutamate receptor activation. Biol. Psychiatry 67, 823–830. 10.1016/j.biopsych.2009.12.018 20132926

[B24] Cippitelli A. Zook M. Bell L. Damadzic R. Eskay R. L. Schwandt M.. (2010b). Reversibility of object recognition but not spatial memory impairment following binge-like alcohol exposure in rats. Neurobiol. Learn. Mem. 94, 538–546. 10.1016/j.nlm.2010.09.006 20849966 PMC2975859

[B25] Cortese B. M. Krahl S. E. Berman R. F. Hannigan J. H. (1997). Effects of prenatal ethanol exposure on hippocampal theta activity in the rat. Alcohol 14, 231–235. 10.1016/s0741-8329(96)00147-4 9160800

[B26] Cudd T. A. (2005). Animal model systems for the study of alcohol teratology. Exp. Biol. Med. 230, 389–393. 10.1177/15353702-0323006-06 15956768

[B27] Davies D. L. Smith D. E. (1981). A Golgi study of mouse hippocampal CA1 pyramidal neurons following perinatal ethanol exposure. Neurosci. Lett. 26, 49–54. 10.1016/0304-3940(81)90424-9 7290537

[B29] Denny L. Coles S. Blitz R. (2017). Fetal alcohol syndrome and fetal alcohol spectrum disorders. Am. Fam. Physician 96, 515–522. 29094891

[B30] Dhanabalan G. Le Maitre T. W. Bogdanovic N. Alkass K. Druid H. (2018). Hippocampal granule cell loss in human chronic alcohol abusers. Neurobiol. Dis. 120, 63–75. 10.1016/j.nbd.2018.08.011 30189262

[B31] Diaz Perez H. Espinosa Villanueva J. Machado Salas J. (1991). Behavioral and hippocampal morphological changes induced by ethanol administered to pregnant rats. Ann. N Y Acad. Sci. 625, 300–304. 10.1111/j.1749-6632.1991.tb33855.x 2058890

[B32] Drissi I. Deschamps C. Fouquet G. Alary R. Peineau S. Gosset P.. (2019). Memory and plasticity impairment after binge drinking in adolescent rat hippocampus: GluN2A/GluN2B NMDA receptor subunits imbalance through HDAC2. Addict. Biol. [Epub ahead of print]. 10.1111/adb.12760 31056842

[B33] Dubreuil D. Tixier C. Dutrieux G. Edeline J. M. (2003). Does the radial arm maze necessarily test spatial memory? Neurobiol. Learn. Mem. 79, 109–117. 10.1016/s1074-7427(02)00023-0 12482685

[B34] Durand D. Saint-Cyr J. A. Gurevich N. Carlen P. L. (1989). Ethanol-induced dendritic alterations in hippocampal granule cells. Brain Res. 477, 373–377. 10.1016/0006-8993(89)91430-3 2467727

[B35] Ehlers C. L. Liu W. Wills D. N. Crews F. T. (2013). Periadolescent ethanol vapor exposure persistently reduces measures of hippocampal neurogenesis that are associated with behavioral outcomes in adulthood. Neuroscience 244, 1–15. 10.1016/j.neuroscience.2013.03.058 23567812 PMC3726260

[B36] Ewin S. E. Morgan J. W. Niere F. McMullen N. P. Barth S. H. Almonte A. G.. (2019). Chronic intermittent ethanol exposure selectively increases synaptic excitability in the ventral domain of the rat hippocampus. Neuroscience 398, 144–157. 10.1016/j.neuroscience.2018.11.028 30481568 PMC6658135

[B37] Fanselow M. S. Dong H. W. (2010). Are the dorsal and ventral hippocampus functionally distinct structures? Neuron 65, 7–19. 10.1016/j.neuron.2009.11.031 20152109 PMC2822727

[B38] Farr S. A. Scherrer J. F. Banks W. A. Flood J. F. Morley J. E. (2005). Chronic ethanol consumption impairs learning and memory after cessation of ethanol. Alcohol. Clin. Exp. Res. 29, 971–982. 10.1097/01.alc.0000171038.03371.56 15976523

[B39] Fedorchak A. V. Miller M. W. (2019). Episodic prenatal exposure to ethanol affects postnatal neurogenesis in the macaque dentate gyrus and visual recognition memory. Int. J. Dev. Neurosci. 79, 65–75. 10.1016/j.ijdevneu.2019.10.005 31706015

[B40] Fehr K. A. Kalant H. LeBlanc A. E. (1976). Residual learning deficit after heavy exposure to cannabis or alcohol in rats. Science 192, 1249–1251. 10.1126/science.1273591 1273591

[B41] Fernandes L. M. P. Cartágenes S. C. Barros M. A. Carvalheiro T. Castro N. C. F. Schamne M. G.. (2018). Repeated cycles of binge-like ethanol exposure induce immediate and delayed neurobehavioral changes and hippocampal dysfunction in adolescent female rats. Behav. Brain Res. 350, 99–108. 10.1016/j.bbr.2018.05.007 29752970

[B42] Ferrer I. Galofré F. López-Tejero D. Llobera M. (1988). Morphological recovery of hippocampal pyramidal neurons in the adult rat exposed *in utero* to ethanol. Toxicology 48, 191–197. 10.1016/0300-483x(88)90100-x 3341045

[B43] Fiala J. C. Spacek J. Harris K. M. (2002). Dendritic spine pathology: cause or consequence of neurological disorders? J. Gerontol. A Biol. Sci. Med. Sci. 39, 29–54. 10.1016/s0165-0173(02)00158-3 12086707

[B44] Fish E. W. Holloway H. T. Rumple A. Baker L. K. Wieczorek L. A. Moy S. S.. (2016). Acute alcohol exposure during neurulation: behavioral and brain structural consequences in adolescent C57BL/6J mice. Behav. Brain Res. 311, 70–80. 10.1016/j.bbr.2016.05.004 27185739 PMC4931949

[B45] Fish E. W. Wieczorek L. A. Rumple A. Suttie M. Moy S. S. Hammond P.. (2018). The enduring impact of neurulation stage alcohol exposure: a combined behavioral and structural neuroimaging study in adult male and female C57BL/6J mice. Behav. Brain Res. 338, 173–184. 10.1016/j.bbr.2017.10.020 29107713 PMC5726510

[B46] Fleming R. L. Acheson S. K. Moore S. D. Wilson W. A. Swartzwelder H. S. (2012). In the rat, chronic intermittent ethanol exposure during adolescence alters the ethanol sensitivity of tonic inhibition in adulthood. Alcohol. Clin. Exp. Res. 36, 279–285. 10.1111/j.1530-0277.2011.01615.x 22014205 PMC3732030

[B47] Fleming R. L. Li Q. Risher M. L. Sexton H. G. Moore S. D. Wilson W. A.. (2013). Binge-pattern ethanol exposure during adolescence, but not adulthood, causes persistent changes in GABA_A_ receptor-mediated tonic inhibition in dentate granule cells. Alcohol. Clin. Exp. Res. 37, 1154–1160. 10.1111/acer.12087 23413887 PMC3754782

[B48] Fontaine C. J. Pinar C. Yang W. Pang A. F. Suesser K. E. Choi J. S. J.. (2019). Impaired bidirectional synaptic plasticity in juvenile offspring following prenatal ethanol exposure. Alcohol. Clin. Exp. Res. 43, 2153–2166. 10.1111/acer.14170 31386206 PMC6779507

[B49] Franke H. Kittner H. Berger P. Wirkner K. Schramek J. (1997). The reaction of astrocytes and neurons in the hippocampus of adult rats during chronic ethanol treatment and correlations to behavioral impairments. Alcohol 14, 445–454. 10.1016/s0741-8329(96)00209-1 9305459

[B50] Gabriel M. (1993). “Discriminative avoidance learning: a model system,” in Neurobiology of Cingulate Cortex and Limbic Thalamus: A Comprehensive Handbook, eds Vogt B. A. Gabriel M. (Toronto: Birkhauser), 478–523.

[B51] Gál K. Bárdos G. (1994). The effect of chronic alcohol treatment on the radial maze performance of rats. Neuroreport 5, 421–424. 10.1097/00001756-199401120-00012 8003666

[B52] Gianoulakis C. (1990). Rats exposed prenatally to alcohol exhibit impairment in spatial navigation test. Behav. Brain Res. 36, 217–228. 10.1016/0166-4328(90)90060-r 2310487

[B53] Gibson W. E. (1985). Effects of alcohol on radial maze performance in rats. Physiol. Behav. 35, 1003–1005. 10.1016/0031-9384(85)90273-2 4095174

[B55] Givens B. (1995). Low-doses of ethanol impair spatial working-memory and reduce hippocampal theta-activity. Alcohol. Clin. Exp. Res. 19, 763–767. 10.1111/j.1530-0277.1995.tb01580.x 7573806

[B54] Givens B. McMahon K. (1995). Ethanol suppresses the induction of long-term potentiation *in vivo*. Brain Res. 688, 27–33. 10.1016/0006-8993(95)00499-g 8542319

[B56] Goeke C. M. Roberts M. L. Hashimoto J. G. Finn D. A. Guizzetti M. (2018). Neonatal ethanol and choline treatments alter the morphology of developing rat hippocampal pyramidal neurons in opposite directions. Neuroscience 374, 13–24. 10.1016/j.neuroscience.2018.01.031 29391132 PMC5835200

[B57] González-Burgos I. Alejandre-Gùmez M. Olvera-Cortés M. E. Perùz-Vega M. I. Evans S. Feria-Velasco A. (2006). Prenatal-through-postnatal exposure to moderate levels of ethanol leads to damage on the hippocampal CA1 field of juvenile rats: a stereology and Golgi study. Neurosci. Res. 56, 400–408. 10.1016/j.neures.2006.08.007 16978724

[B58] Guzmán-Vélez E. Warren D. E. Feinstein J. S. Bruss J. Tranel D. (2016). Dissociable contributions of amygdala and hippocampus to emotion and memory in patients with Alzheimer’s disease. Hippocampus 26, 727–738. 10.1002/hipo.22554 26606553

[B59] Harper C. (1998). The neuropathology of alcohol-specific brain damage, or does alcohol damage the brain? Brain Behav. Immun. 57, 101–110. 10.1097/00005072-199802000-00001 9600202

[B60] Harris K. M. Kater S. B. (1994). Dendritic spines: cellular specializations imparting both stability and flexibility to synaptic function. Annu. Rev. Neurosci. 17, 341–371. 10.1146/annurev.ne.17.030194.002013 8210179

[B61] Harvey R. J. Skelton-Robinson M. Rossor M. N. (2003). The prevalence and causes of dementia in people under the age of 65 years. J. Neurol. Neurosurg. Psychiatry 74, 1206–1209. 10.1136/jnnp.74.9.1206 12933919 PMC1738690

[B62] Helfer J. L. White E. R. Christie B. R. (2012). Enhanced deficits in long-term potentiation in the adult dentate gyrus with 2nd trimester ethanol consumption. PLoS One 7:e51344. 10.1371/journal.pone.0051344 23227262 PMC3515437

[B63] Hendricson A. W. Sibbald J. R. Morrisett R. A. (2004). Ethanol alters the frequency, amplitude, and decay kinetics of Sr^2+^-supported, asynchronous NMDAR mEPSCs in rat hippocampal slices. J. Neurophysiol. 91, 2568–2577. 10.1152/jn.00997.2003 14749312

[B64] Heroux N. A. Robinson-Drummer P. A. Kawan M. Rosen J. B. Stanton M. E. (2019). Neonatal ethanol exposure impairs long-term context memory formation and prefrontal immediate early gene expression in adolescent rats. Behav. Brain Res. 359, 386–395. 10.1016/j.bbr.2018.11.018 30447241 PMC6314039

[B65] Hoff S. F. (1988). Synaptogenesis in the hippocampal dentate gyrus: effects of *in utero* ethanol exposure. Brain Res. Bull. 21, 47–54. 10.1016/0361-9230(88)90119-0 3219600

[B66] Ieraci A. Herrera D. G. (2007). Single alcohol exposure in early life damages hippocampal stem/progenitor cells and reduces adult neurogenesis. Neurobiol. Dis. 26, 597–605. 10.1016/j.nbd.2007.02.011 17490887

[B67] Ikonomidou C. Bittigau P. Ishimaru M. J. Wozniak D. F. Koch C. Genz K.. (2000). Ethanol-induced apoptotic neurodegeneration and fetal alcohol syndrome. Science 287, 1056–1060. 10.1126/science.287.5455.1056 10669420

[B68] Izumi Y. Nagashima K. Murayama K. Zorumski C. F. (2005). Acute effects of ethanol on hippocampal long-term potentiation and long-term depression are mediated by different mechanisms. Neuroscience 136, 509–517. 10.1016/j.neuroscience.2005.08.002 16216426

[B69] Jakubowska-Dogru E. Elibol B. Dursun I. Yüruker S. (2017). Effects of prenatal binge-like ethanol exposure and maternal stress on postnatal morphological development of hippocampal neurons in rats. Int. J. Dev. Neurosci. 61, 40–50. 10.1016/j.ijdevneu.2017.06.002 28636875

[B70] Jang M. H. Shin M. C. Jung S. B. Lee T. H. Bahn G. H. Kwon Y. K.. (2002). Alcohol and nicotine reduce cell proliferation and enhance apoptosis in dentate gyrus. Neuroreport 13, 1509–1513. 10.1097/00001756-200208270-00004 12218695

[B71] Jernigan T. L. Butters N. Ditraglia G. Schafer K. Smith T. Irwin M.. (1991). Reduced cerebral grey matter observed in alcoholics using magnetic resonance imaging. Alcohol. Clin. Exp. Res. 15, 418–427. 10.1111/j.1530-0277.1991.tb00540.x 1877728

[B72] Johnsen-Soriano S. Bosch-Morell F. Miranda M. Asensio S. Barcia J. M. Roma J.. (2007). Ebselen prevents chronic alcohol-induced rat hippocampal stress and functional impairment. Alcohol. Clin. Exp. Res. 31, 486–492. 10.1111/j.1530-0277.2006.00329.x 17295734

[B74] Kim J. J. Jung M. W. (2006). Neural circuits and mechanisms involved in Pavlovian fear conditioning: a critical review. Neurosci. Biobehav. Rev. 30, 188–202. 10.1016/j.neubiorev.2005.06.005 16120461 PMC4342048

[B75] King M. A. Hunter B. E. Walker D. W. (1988). Alterations and recovery of dendritic spine density in rat hippocampus following long-term ethanol ingestion. Brain Res. 459, 381–385. 10.1016/0006-8993(88)90656-7 3179712

[B76] Klintsova A. Y. Helfer J. L. Calizo L. H. Dong W. K. Goodlett C. R. Greenough W. T. (2007). Persistent impairment of hippocampal neurogenesis in young adult rats following early postnatal alcohol exposure. Alcohol. Clin. Exp. Res. 31, 2073–2082. 10.1111/j.1530-0277.2007.00528.x 17949464

[B77] Koob G. F. Le Moal M. (2005). Plasticity of reward neurocircuitry and the ‘dark side’ of drug addiction. Nat. Neurosci. 8, 1442–1444. 10.1038/nn1105-1442 16251985

[B78] Kuntsche E. Knibbe R. Gmel G. Engels R. (2005). Why do young people drink? A review of drinking motives. Clin. Psychol. Rev. 25, 841–861. 10.1016/j.cpr.2005.06.002 16095785

[B79] Le Maître T. W. Dhanabalan G. Bogdanovic N. Alkass K. Druid H. (2018). Effects of alcohol abuse on proliferating cells, stem/progenitor cells and immature neurons in the adult human hippocampus. Neuropsychopharmacology 43, 690–699. 10.1038/npp.2017.251 29052615 PMC5809795

[B81] Lee K. Dunwiddie T. Deitrich R. Lynch G. Hoffer B. (1981). Chronic ethanol-consumption and hippocampal neuron dendritic spines—a morphometric and physiological analysis. Exp. Neurol. 71, 541–549. 10.1016/0014-4886(81)90031-5 7461079

[B80] Lee D. H. Moon J. Ryu J. Jeong J. Y. Roh G. S. Kim H. J.. (2016). Effects of postnatal alcohol exposure on hippocampal gene expression and learning in adult mice. Genes Genet. Syst. 90, 335–342. 10.1266/ggs.15-00026 26960969

[B83] Lescaudron L. Jaffard R. Verna A. (1989). Modifications in number and morphology of dendritic spines resulting from chronic ethanol-consumption and withdrawal—a Golgi-study in the mouse anterior and posterior hippocampus. Exp. Neurol. 106, 156–163. 10.1016/0014-4886(89)90089-7 2478383

[B82] Lescaudron L. Verna A. (1985). Effects of chronic ethanol-consumption on pyramidal neurons of the mouse dorsal and ventral hippocampus—a quantitative histological analysis. Exp. Brain Res. 58, 362–367. 10.1007/bf00235317 4039674

[B84] Li Q. Fleming R. L. Acheson S. K. Madison R. D. Moore S. D. Risher M. L.. (2013). Long-term modulation of A-type K^+^ conductances in hippocampal CA1 interneurons in rats after chronic intermittent ethanol exposure during adolescence or adulthood. Alcohol. Clin. Exp. Res. 37, 2074–2085. 10.1111/acer.12204 23889304 PMC4088965

[B85] Livy D. J. Miller E. K. Maier S. E. West J. R. (2003). Fetal alcohol exposure and temporal vulnerability: effects of binge-like alcohol exposure on the developing rat hippocampus. Neurotoxicol. Teratol. 25, 447–458. 10.1016/s0892-0362(03)00030-8 12798962

[B86] Lobaugh N. J. Wigal T. Greene P. L. Diaz-Granados J. L. Amsel A. (1991). Effects of prenatal ethanol exposure on learned persistence and hippocampal neuroanatomy in infant, weanling and adult rats. Behav. Brain Res. 44, 81–86. 10.1016/s0166-4328(05)80241-4 1910573

[B87] Ludvig N. Altura B. T. Fox S. E. Altura B. M. (1995). The suppressant effect of ethanol, delivered *via* intra hippocampal microdialysis, on the firing of local pyramidal cells in freely behaving rats. Alcohol 12, 417–421. 10.1016/0741-8329(95)00012-g 8519436

[B88] Lukoyanov N. V. Brandão F. Cadete-Leite A. Madeira M. D. Paula-Barbosa M. M. (2000). Synaptic reorganization in the hippocampal formation of alcohol-fed rats may compensate for functional deficits related to neuronal loss. Alcohol 20, 139–148. 10.1016/s0741-8329(99)00069-5 10719793

[B89] Lundqvist C. Alling C. Knoth R. Volk B. (1995). Intermittent ethanol exposure of adult rats: hippocampal cell loss after one month of treatment. Alcohol Alcohol. 30, 737–748. 8679014

[B90] Maier S. E. West J. R. (2001). Regional differences in cell loss associated with binge-like alcohol exposure during the first two trimesters equivalent in the rat. Alcohol 23, 49–57. 10.1016/s0741-8329(00)00133-6 11282452

[B91] Mameli M. Zamudio P. A. Carta M. Valenzuela C. F. (2005). Developmentally regulated actions of alcohol on hippocampal glutamatergic transmission. J. Neurosci. 25, 8027–8036. 10.1523/JNEUROSCI.2434-05.2005 16135760 PMC6725449

[B92] Markwiese B. J. Acheson S. K. Levin E. D. Wilson W. A. Swartzwelder H. S. (1998). Differential effects of ethanol on memory in adolescent and adult rats. Alcohol. Clin. Exp. Res. 22, 416–421. 10.1111/j.1530-0277.1998.tb03668.x 9581648

[B93] Matthews D. B. Simson P. E. (1998). Prenatal exposure to ethanol disrupts spatial memory: effect of the training-testing delay period. Physiol. Behav. 64, 63–67. 10.1016/s0031-9384(98)00019-5 9661983

[B94] Matthews D. B. Simson P. E. Best P. J. (1995). Acute ethanol impairs spatial memory but not stimulus-response memory in the rat. Alcohol. Clin. Exp. Res. 19, 902–909. 10.1111/j.1530-0277.1995.tb00965.x 7485837

[B95] Matthews D. B. Simson P. E. Best P. J. (1996). Ethanol alters spatial processing of hippocampal place cells: a mechanism for impaired navigation when intoxicated. Alcohol. Clin. Exp. Res. 20, 404–407. 10.1111/j.1530-0277.1996.tb01660.x 8730237

[B96] McClain J. A. Morris S. A. Deeny M. A. Marshall S. A. Hayes D. M. Kiser Z. M.. (2011). Adolescent binge alcohol exposure induces long-lasting partial activation of microglia. Brain Behav. Immun. 25, S120–S128. 10.1016/j.bbi.2011.01.006 21262339 PMC3098298

[B97] McClain J. A. Morris S. A. Marshall S. A. Nixon K. (2014). Ectopic hippocampal neurogenesis in adolescent male rats following alcohol dependence. Addict. Biol. 19, 687–699. 10.1111/adb.12075 23844726 PMC3844012

[B98] McMullen P. A. Stcyr-Cyr J. A. Carlen P. L. (1984). Morphological alterations in rat Ca1 hippocampal pyramidal cell dendrites resulting from chronic ethanol-consumption and withdrawal. J. Comp. Neurol. 225, 111–118. 10.1002/cne.902250112 6539344

[B99] Melia K. R. Ryabinin A. E. Corodimas K. P. Wilson M. C. Ledoux J. E. (1996). Hippocampal-dependent learning and experience-dependent activation of the hippocampus are preferentially disrupted by ethanol. Neuroscience 74, 313–322. 10.1016/0306-4522(96)00138-8 8865184

[B100] Merrill J. E. Carey K. B. (2016). Drinking over the lifespan: focus on college ages. Alcohol. Res. 38, 103–114. 27159817 10.35946/arcr.v38.1.13 PMC4872605

[B101] Miller M. W. (1995). Generation of neurons in the rat dentate gyrus and hippocampus: effects of prenatal and postnatal treatment with ethanol. Alcohol. Clin. Exp. Res. 19, 1500–1509. 10.1111/j.1530-0277.1995.tb01014.x 8749817

[B73] Moriyama Y. Mimura M. Kato M. Kashima H. (2006). Primary alcoholic dementia and alcohol-related dementia. Psicogeriatrics 6, 114–118. 10.1111/j.1479-8301.2006.00168.x

[B102] Morris R. (1984). Developments of a water-maze procedure for studying spatial learning in the rat. J. Neurosci. Methods 11, 47–60. 10.1016/0165-0270(84)90007-4 6471907

[B103] Morris S. A. Eaves D. W. Smith A. R. Nixon K. (2010). Alcohol inhibition of neurogenesis: a mechanism of hippocampal neurodegeneration in an adolescent alcohol abuse model. Hippocampus 20, 596–607. 10.1002/hipo.20665 19554644 PMC2861155

[B104] Morrisett R. A. Swartzwelder H. S. (1993). Attenuation of hippocampal long-term potentiation by ethanol: a patch-clamp analysis of glutamatergic and GABAergic mechanisms. J. Neurosci. 13, 2264–2272. 10.1523/JNEUROSCI.13-05-02264.1993 8478698 PMC6576561

[B105] Moser M. B. Moser E. I. (1998). Functional differentiation in the hippocampus. Hippocampus 8, 608–619. 10.1002/(sici)1098-1063(1998)8:6<608::aid-hipo3>3.0.co;2-7 9882018

[B106] Moser M. B. Moser E. I. Forrest E. Andersen P. Morris R. G. (1995). Spatial learning with a minislab in the dorsal hippocampus. Proc. Natl. Acad. Sci. U S A 92, 9697–9701. 10.1073/pnas.92.21.9697 7568200 PMC40869

[B107] Mulholland P. J. Teppen T. L. Miller K. M. Sexton H. G. Pandey S. C. Swartzwelder H. S. (2018). Donepezil reverses dendritic spine morphology adaptations and Fmr1 epigenetic modifications in hippocampus of adult rats after adolescent alcohol exposure. Alcohol. Clin. Exp. Res. 42, 706–717. 10.1111/acer.13599 29336496 PMC5903436

[B108] Nelson T. E. Ur C. L. Gruol D. L. (2005). Chronic intermittent ethanol exposure enhances NMDA-receptor-mediated synaptic responses and NMDA receptor expression in hippocampal CA1 region. Brain Res. 1048, 69–79. 10.1016/j.brainres.2005.04.041 15919065

[B109] Obernier J. A. Bouldin T. W. Crews F. T. (2002). Binge ethanol exposure in adult rats causes necrotic cell death. Alcohol. Clin. Exp. Res. 26, 547–557. 10.1111/j.1530-0277.2002.tb02573.x 11981132

[B110] Olateju O. I. Spocter M. A. Patzke N. Ihunwo A. O. Manger P. R. (2018). Hippocampal neurogenesis in the C57BL/6J mice at early adulthood following prenatal alcohol exposure. Metab. Brain Dis. 33, 397–410. 10.1007/s11011-017-0156-4 29164372

[B111] Olton D. S. Samuelson R. J. (1976). Remembrance of places passed—spatial memory in rats. J. Exp. Psychol. Anim. Behav. Process. 2, 97–116. 10.1037/0097-7403.2.2.97

[B112] Oshiro W. M. Beasley T. E. McDaniel K. L. Taylor M. M. Evansky P. Moser V. C.. (2014). Selective cognitive deficits in adult rats after prenatal exposure to inhaled ethanol. Neurotoxicol. Teratol. 45, 44–58. 10.1016/j.ntt.2014.07.001 25020118

[B113] Patten A. R. Brocardo P. S. Sakiyama C. Wortman R. C. Noonan A. Gil-Mohapel J.. (2013). Impairments in hippocampal synaptic plasticity following prenatal ethanol exposure are dependent on glutathione levels. Hippocampus 23, 1463–1475. 10.1002/hipo.22199 23996467

[B114] Paula-Barbosa M. M. Brandão F. Madeira M. D. Cadete-Leite A. (1993). Structural changes in the hippocampal formation after long-term alcohol consumption and withdrawal in the rat. Addiction 88, 237–247. 10.1111/j.1360-0443.1993.tb00807.x 8220061

[B115] Phillips S. C. Cragg B. G. (1983). Chronic consumption of alcohol by adult mice—effect on hippocampal cells and synapses. Exp. Neurol. 80, 218–226. 10.1016/0014-4886(83)90018-3 6682045

[B116] Pothuizen H. H. Zhang W. N. Jongen-Rêlo A. L. Feldon J. Yee B. K. (2004). Dissociation of function between the dorsal and the ventral hippocampus in spatial learning abilities of the rat: a within-subject, within-task comparison of reference and working spatial memory. Eur. J. Neurosci. 19, 705–712. 10.1111/j.0953-816x.2004.03170.x 14984421

[B117] Poucet B. Benhamou S. (1997). The neuropsychology of spatial cognition in the rat. Crit. Rev. Neurobiol. 11, 101–120. 10.1615/critrevneurobiol.v11.i2-3.10 9209826

[B118] Puglia M. P. Valenzuela C. F. (2010). Repeated third trimester-equivalent ethanol exposure inhibits long-term potentiation in the hippocampal CA1 region of neonatal rats. Alcohol 44, 283–290. 10.1016/j.alcohol.2010.03.001 20488644 PMC2916030

[B119] Qin L. Crews F. T. (2012). NADPH oxidase and reactive oxygen species contribute to alcohol-induced microglial activation and neurodegeneration. J. Neuroinflammation 9:5. 10.1186/1742-2094-9-5 22240163 PMC3271961

[B120] Rebola N. Carta M. Mulle C. (2017). Operation and plasticity of hippocampal CA3 circuits: implications for memory encoding. Nat. Rev. Neurosci. 18, 208–220. 10.1038/nrn.2017.10 28251990

[B121] Ridley N. J. Draper B. Withall A. (2013). Alcohol-related dementia: an update of the evidence. Alzheimers Res. Ther. 5:3. 10.1186/alzrt157 23347747 PMC3580328

[B122] Riley J. N. Walker D. W. (1978). Morphological alterations in hippocampus after long-term alcohol consumption in mice. Science 201, 646–648. 10.1126/science.566953 566953

[B123] Risher M. L. Fleming R. L. Risher W. C. Miller K. M. Klein R. C. Wills T.. (2015). Adolescent intermittent alcohol exposure: persistence of structural and functional hippocampal abnormalities into adulthood. Alcohol. Clin. Exp. Res. 39, 989–997. 10.1111/acer.12725 25916839 PMC4452443

[B124] Roberto M. Nelson T. E. Ur C. L. Gruol D. L. (2002). Long-term potentiation in the rat hippocampus is reversibly depressed by chronic intermittent ethanol exposure. J. Neurophysiol. 87, 2385–2397. 10.1152/jn.2002.87.5.2385 11976376

[B125] Sadrian B. Lopez-Guzman M. Wilson D. A. Saito M. (2014). Distinct neurobehavioral dysfunction based on the timing of developmental binge-like alcohol exposure. Neuroscience 280, 204–219. 10.1016/j.neuroscience.2014.09.008 25241068 PMC4250396

[B126] Sanchez L. M. Goss J. Wagner J. Davies S. Savage D. D. Hamilton D. A.. (2019). Moderate prenatal alcohol exposure impairs performance by adult male rats in an object-place paired-associate task. Behav. Brain Res. 360, 228–234. 10.1016/j.bbr.2018.12.014 30529401 PMC6324964

[B127] Santangelo V. Cavallina C. Colucci P. Santori A. Macri S. Mcgaugh J. L.. (2018). Enhanced brain activity associated with memory access in highly superior autobiographical memory. Proc. Natl. Acad. Sci. U S A 115, 7795–7800. 10.1073/pnas.1802730115 29987025 PMC6064994

[B128] Schulteis G. Archer C. Tapert S. F. Frank L. R. (2008). Intermittent binge alcohol exposure during the periadolescent period induces spatial working memory deficits in young adult rats. Alcohol 42, 459–467. 10.1016/j.alcohol.2008.05.002 18760715 PMC2562042

[B129] Shield K. D. Parry C. Rehm J. (2013). Chronic diseases and conditions related to alcohol use. Alcohol Res. 35, 155–173. 24881324 10.35946/arcr.v35.2.06 PMC3908707

[B130] Shimizu K. Matsubara K. Uezono T. Kimura K. Shiono H. (1998). Reduced dorsal hippocampal glutamate release significantly correlates with the spatial memory deficits produced by benzodiazepines and ethanol. Neuroscience 83, 701–706. 10.1016/s0306-4522(97)00339-4 9483554

[B131] Sickmann H. M. Patten A. R. Morch K. Sawchuk S. Zhang C. Parton R.. (2014). Prenatal ethanol exposure has sex-specific effects on hippocampal long-term potentiation. Hippocampus 24, 54–64. 10.1002/hipo.22203 23996604

[B28] Silvestre de Ferron B. Bennouar K. E. Kervern M. Alaux-Cantin S. Robert A. Rabiant K.. (2016). Two binges of ethanol a day keep the memory away in adolescent rats: key role for GLUN2B subunit. Int. J. Neuropsychopharmacol. 19:pyv087. 10.1093/ijnp/pyv087 26254123 PMC4772273

[B132] Simson P. E. Criswell H. E. Breese G. R. (1993). Inhibition of NMDA-evoked electrophysiological activity by ethanol in selected brain regions: evidence for ethanol-sensitive and ethanol-insensitive NMDA-evoked responses. Brain Res. 607, 9–16. 10.1016/0006-8993(93)91483-9 8481813

[B133] Sinclair J. G. Lo G. F. (1986). Ethanol blocks tetanic and calcium-induced long-term potentiation in the hippocampal slice. Gen. Pharmacol. 17, 231–233. 10.1016/0306-3623(86)90144-8 3699450

[B135] Sircar R. Basak A. K. Sircar D. (2009). Repeated ethanol exposure affects the acquisition of spatial memory in adolescent female rats. Behav. Brain Res. 202, 225–231. 10.1016/j.bbr.2009.03.036 19463705 PMC2694351

[B134] Sircar R. Sircar D. (2005). Adolescent rats exposed to repeated ethanol treatment show lingering behavioral impairments. Alcohol. Clin. Exp. Res. 29, 1402–1410. 10.1097/01.alc.0000175012.77756.d9 16131847

[B136] Sloan F. Grossman D. Platt A. (2011). Heavy episodic drinking in early adulthood and outcomes in midlife. J. Stud. Alcohol Drugs 72, 459–470. 10.15288/jsad.2011.72.459 21513683 PMC3084361

[B137] Staples M. C. Kim A. Mandyam C. D. (2015). Dendritic remodeling of hippocampal neurons is associated with altered NMDA receptor expression in alcohol dependent rats. Mol. Cell. Neurosci. 65, 153–162. 10.1016/j.mcn.2015.03.008 25769285 PMC4395499

[B138] Steffensen S. C. Henriksen S. J. (1992). Comparison of the effects of ethanol and chlordiazepoxide on electrophysiological activity in the fascia dentata and hippocampus regio superior. Hippocampus 2, 201–211. 10.1002/hipo.450020210 1308183

[B139] Steffensen S. C. Yeckel M. F. Miller D. R. Henriksen S. J. (1993). Ethanol-induced suppression of hippocampal long-term potentiation is blocked by lesions of the septohippocampal nucleus. Alcohol. Clin. Exp. Res. 17, 655–659. 10.1111/j.1530-0277.1993.tb00814.x 8333597

[B140] Stella F. Cerasti E. Si B. Jezek K. Treves A. (2012). Self-organization of multiple spatial and context memories in the hippocampus. Neurosci. Biobehav. Rev. 36, 1609–1625. 10.1016/j.neubiorev.2011.12.002 22192880

[B141] Stephens D. N. Ripley T. L. Borlikova G. Schubert M. Albrecht D. Hogarth L.. (2005). Repeated ethanol exposure and withdrawal impairs human fear conditioning and depresses long-term potentiation in rat amygdala and hippocampus. Biol. Psychiatry 58, 392–400. 10.1016/j.biopsych.2005.04.025 16018978

[B142] Stragier E. Martin V. Davenas E. Poilbout C. Mongeau R. Corradetti R.. (2015). Brain plasticity and cognitive functions after ethanol consumption in C57BL/6J mice. Transl. Psychiatry 5:e696. 10.1038/tp.2015.183 26670281 PMC5068583

[B143] Stuchlik A. (2014). Dynamic learning and memory, synaptic plasticity and neurogenesis: an update. Front. Behav. Neurosci. 8:106. 10.3389/fnbeh.2014.00106 24744707 PMC3978286

[B144] Sutherland R. J. Mcdonald R. J. Savage D. D. (1997). Prenatal exposure to moderate levels of ethanol can have long-lasting effects on hippocampal synaptic plasticity in adult offspring. Hippocampus 7, 232–238. 10.1002/(sici)1098-1063(1997)7:2<232::aid-hipo9>3.0.co;2-o 9136052

[B145] Swartzwelder H. S. Farr K. L. Wilson W. A. Savage D. D. (1988). Prenatal exposure to ethanol decreases physiological plasticity in the hippocampus of the adult-rat. Alcohol 5, 121–124. 10.1016/0741-8329(88)90008-0 3395460

[B146] Swartzwelder H. S. Risher M. L. Miller K. M. Colbran R. J. Winder D. G. Wills T. A. (2016). Changes in the adult GluN2B associated proteome following adolescent intermittent ethanol exposure. PLoS One 11:e0155951. 10.1371/journal.pone.0155951 27213757 PMC4877005

[B147] Swartzwelder H. S. Wilson W. A. Tayyeb M. I. (1995a). Age-dependent inhibition of long-term potentiation by ethanol in immature versus mature hippocampus. Alcohol. Clin. Exp. Res. 19, 1480–1485. 10.1111/j.1530-0277.1995.tb01011.x 8749814

[B148] Swartzwelder H. S. Wilson W. A. Tayyeb M. I. (1995b). Differential sensitivity of Nmda receptor-mediated synaptic potentials to ethanol in immature versus mature hippocampus. Alcohol. Clin. Exp. Res. 19, 320–323. 10.1111/j.1530-0277.1995.tb01509.x 7625564

[B149] Tapia-Rojas C. Carvajal F. J. Mira R. G. Arce C. Lerma-Cabrera J. M. Orellana J. A.. (2018). Adolescent binge alcohol exposure affects the brain function through mitochondrial impairment. Mol. Neurobiol. 55, 4473–4491. 10.1007/s12035-017-0613-4 28674997

[B150] Tapia-Rojas C. Mira R. G. Torres A. K. Jara C. Pérez M. J. Vergara E. H.. (2017). Alcohol consumption during adolescence: a link between mitochondrial damage and ethanol brain intoxication. Birth Defects Res. 109, 1623–1639. 10.1002/bdr2.1172 29251843

[B151] Tarelo-Acuña L. Olvera-Cortés E. González-Burgos I. (2000). Prenatal and postnatal exposure to ethanol induces changes in the shape of the dendritic spines from hippocampal CA1 pyramidal neurons of the rat. Neurosci. Lett. 286, 13–16. 10.1016/s0304-3940(00)01075-2 10822141

[B152] Titterness A. K. Christie B. R. (2012). Prenatal ethanol exposure enhances NMDAR-dependent long-term potentiation in the adolescent female dentate gyrus. Hippocampus 22, 69–81. 10.1002/hipo.20849 21080406

[B153] Tokuda K. Zorumski C. F. Izumi Y. (2007). Modulation of hippocampal long-term potentiation by slow increases in ethanol concentration. Neuroscience 146, 340–349. 10.1016/j.neuroscience.2007.01.037 17346891 PMC1934937

[B154] Toyoda H. Li X. Y. Wu L. J. Zhao M. G. Descalzi G. Chen T.. (2011). Interplay of amygdala and cingulate plasticity in emotional fear. Neural Plast. 2011:813749. 10.1155/2011/813749 21912749 PMC3168900

[B155] Uban K. A. Sliwowska J. H. Lieblich S. Ellis L. A. Yu W. K. Weinberg J.. (2010). Prenatal alcohol exposure reduces the proportion of newly produced neurons and glia in the dentate gyrus of the hippocampus in female rats. Horm. Behav. 58, 835–843. 10.1016/j.yhbeh.2010.08.007 20736015 PMC3132584

[B156] Van Skike C. E. Novier A. Diaz-Granados J. L. Matthews D. B. (2012). The effect of chronic intermittent ethanol exposure on spatial memory in adolescent rats: the dissociation of metabolic and cognitive tolerances. Brain Res. 1453, 34–39. 10.1016/j.brainres.2012.03.006 22464184

[B157] Varaschin R. K. Rosenberg M. J. Hamilton D. A. Savage D. D. (2014). Differential effects of the histamine H_3_ receptor agonist methimepip on dentate granule cell excitability, paired-pulse plasticity and long-term potentiation in prenatal alcohol-exposed rats. Alcohol. Clin. Exp. Res. 38, 1902–1911. 10.1111/acer.12430 24818819 PMC5094461

[B158] Vedder L. C. Hall J. M. Jabrouin K. R. Savage L. M. (2015). Interactions between chronic ethanol consumption and thiamine deficiency on neural plasticity, spatial memory, and cognitive flexibility. Alcohol. Clin. Exp. Res. 39, 2143–2153. 10.1111/acer.12859 26419807 PMC4624484

[B159] Vetreno R. P. Crews F. T. (2015). Binge ethanol exposure during adolescence leads to a persistent loss of neurogenesis in the dorsal and ventral hippocampus that is associated with impaired adult cognitive functioning. Front. Neurosci. 9:35. 10.3389/fnins.2015.00035 25729346 PMC4325907

[B160] Wakita M. Shin M. C. Iwata S. Nonaka K. Akaike N. (2012). Effects of ethanol on GABA_A_ receptors in GABAergic and glutamatergic presynaptic nerve terminals. J. Pharmacol. Exp. Ther. 341, 809–819. 10.1124/jpet.111.189126 22434676

[B161] Walker D. W. Barnes D. E. Zornetzer S. F. Hunter B. E. Kubanis P. (1980). Neuronal loss in hippocampus induced by prolonged ethanol consumption in rats. Science 209, 711–713. 10.1126/science.7394532 7394532

[B162] Wang X. Yu H. You J. Wang C. Feng C. Liu Z.. (2018). Memantine can improve chronic ethanol exposure-induced spatial memory impairment in male C57BL/6 mice by reducing hippocampal apoptosis. Toxicology 406–407, 21–32. 10.1016/j.tox.2018.05.013 29800586

[B163] Weiner J. L. Zhang L. Carlen P. L. (1994). Potentiation of GABA_A_-mediated synaptic current by ethanol in hippocampal CA1 neurons—possible role of protein-kinase-C. J. Pharmacol. Exp. Ther. 268, 1388–1395. 10.1016/0006-8993(94)91352-8 8138953

[B164] West J. R. Hamre K. M. Cassell M. D. (1986). Effects of ethanol exposure during the third trimester equivalent on neuron number in rat hippocampus and dentate gyrus. Alcohol. Clin. Exp. Res. 10, 190–197. 10.1111/j.1530-0277.1986.tb05070.x 3521377

[B165] West J. R. Hodges C. A. Black A. C. Jr. (1981). Prenatal exposure to ethanol alters the organization of hippocampal mossy fibers in rats. Science 211, 957–959. 10.1126/science.7466371 7466371

[B166] West R. K. Wooden J. I. Barton E. A. Leasure J. L. (2019). Recurrent binge ethanol is associated with significant loss of dentate gyrus granule neurons in female rats despite concomitant increase in neurogenesis. Neuropharmacology 148, 272–283. 10.1016/j.neuropharm.2019.01.016 30659841

[B167] White A. M. Simson P. E. Best P. J. (1997). Comparison between the effects of ethanol and diazepam on spatial working memory in the rat. Psychopharmacology 133, 256–261. 10.1007/s002130050399 9361331

[B168] WHO (2014). Global Status Report on Alcohol and Health. Geneva: World Health Organization.

[B169] Wilhoit L. F. Scott D. A. Simecka B. A. (2017). Fetal alcohol spectrum disorders: characteristics, complications, and treatment. Community Ment. Health J. 53, 711–718. 10.1007/s10597-017-0104-0 28168434

[B170] Wills T. A. Baucum A. J. II. Holleran K. M. Chen Y. Pasek J. G. Delpire E.. (2017). Chronic intermittent alcohol disrupts the GluN2B-associated proteome and specifically regulates group I mGlu receptor-dependent long-term depression. Addict. Biol. 22, 275–290. 10.1111/adb.12319 26549202 PMC4860359

[B171] Zhao Y. N. Wang F. Fan Y. X. Ping G. F. Yang J. Y. Wu C. F. (2013). Activated microglia are implicated in cognitive deficits, neuronal death, and successful recovery following intermittent ethanol exposure. Behav. Brain Res. 236, 270–282. 10.1016/j.bbr.2012.08.052 22985845

[B172] Zink M. Ferbert T. Frank S. T. Seufert P. Gebicke-Haerter P. J. Spanagel R. (2011). Perinatal exposure to alcohol disturbs spatial learning and glutamate transmission-related gene expression in the adult hippocampus. Eur. J. Neurosci. 34, 457–468. 10.1111/j.1460-9568.2011.07776.x 21722212

